# Combining exercise and nanoparticle-based therapies: a review of physiological synergies and pharmacological potential

**DOI:** 10.3389/fphar.2025.1679552

**Published:** 2026-01-12

**Authors:** Qinhai Wang, Jinxiang Sun, Zhanguo Su, Lijuan Xiang

**Affiliations:** 1 Institute of Sports, Huaqiao University, Quanzhou, Fujian, China; 2 Department of Sport Convergence, Namseoul University, Cheonan, Republic of Korea; 3 Faculty of Physical Education, Huainan Normal University, Huainan, Anhui, China; 4 International College, Krirk University, Bangkok, Thailand; 5 Chongqing Preschool Education College, Chongqing, China

**Keywords:** nanoparticle, exercise, health benefits, oxidative stress, antioxidants

## Abstract

The convergence of nanotechnology and exercise science represents a novel pharmacological approach to enhance therapeutic efficacy, physiological resilience, and performance outcomes. Exercise is known to induce systemic adaptations—such as improved mitochondrial biogenesis, enhanced antioxidant defense, and neuroplasticity—while nanoparticles offer advanced pharmacokinetic profiles, including targeted delivery, controlled release, and increased bioavailability. Recent preclinical studies suggest that combining nanoparticle-based interventions with structured physical activity may produce synergistic effects that surpass the benefits of either strategy alone. This review critically examines the underlying molecular and cellular mechanisms involved in these interactions, with a focus on oxidative stress modulation, mitochondrial function, and neuroprotective pathways. Evidence from animal models highlights improvements in endurance, muscle regeneration, and cognitive preservation when bioactive compounds (e.g., resveratrol, curcumin, iron) are delivered via nanoparticle formulations. Notably, nano-encapsulation enhances pharmacodynamic outcomes compared to conventional delivery systems. Despite these promising findings, clinical translation remains limited, underscoring the urgent need for human trials to determine safety, optimal dosing, and sex-specific responses. Emerging directions include the integration of wearable biosensors for real-time monitoring, personalized exercise-nanotherapy protocols, and applications in neurodegenerative and metabolic disorders. Bridging nanomedicine and exercise pharmacology may unlock new pathways for precision health interventions.

## Introduction

The recent advancements in nanotechnology have created new opportunities in health and medicine, resulting in innovative methods for improving physical performance, tissue repair, and overall wellness (Peña et al., 2022). Nanoparticles have special physicochemical characteristics that show great promise for a range of preventative and therapeutic uses (Koksharov et al., 2022; Musielak et al., 2022). The characteristics include molecular-level interactions with biological systems, offering advantages like improved bioavailability, targeted delivery, and regulated release of active ingredients (Koksharov et al., 2022). The advantages of exercise on human health are well-documented, including enhancements in cardiovascular health, mental wellbeing, and cellular repair (Qiu et al., 2023). These benefits may be affected by several factors, like the frequency, intensity, and duration of the exercise. The combination of exercise with nanoparticle-based interventions appears to yield synergistic effects, enhancing the health benefits of both approaches (Liu et al., 2024).

Nanoparticles gained considerable interest owing to their ability to interact with biological tissues in manners unattainable by macroscopic materials. Nanoparticles can be engineered to transport therapeutic agents, enhance cellular uptake, and enable drug delivery with greater efficiency and precision compared to conventional delivery systems (Xia et al., 2023; Forest and Pourchez, 2022; Li et al., 2022). Furthermore, nanoparticles can selectively target specific organs or tissues, which reduces side effects and enhances therapeutic efficacy (Xia et al., 2023). Gold, silver, and silica nanoparticles have antioxidant, anti-inflammatory, and anti-apoptotic features that can prevent exercise-induced inflammation, muscle damage, and oxidative stress (Yang et al., 2022; Awad et al., 2021; Ziaolhagh et al., 2024; Pinho et al., 2022; Toriumi et al., 2021). Nanoparticles have the potential to enhance exercise effects by reducing muscle inflammation, promoting recovery, and improving muscle tissue adaptation to training stimuli, ultimately leading to enhanced performance over time (Pinho et al., 2022; Toriumi et al., 2021).

**FIGURE 1:**
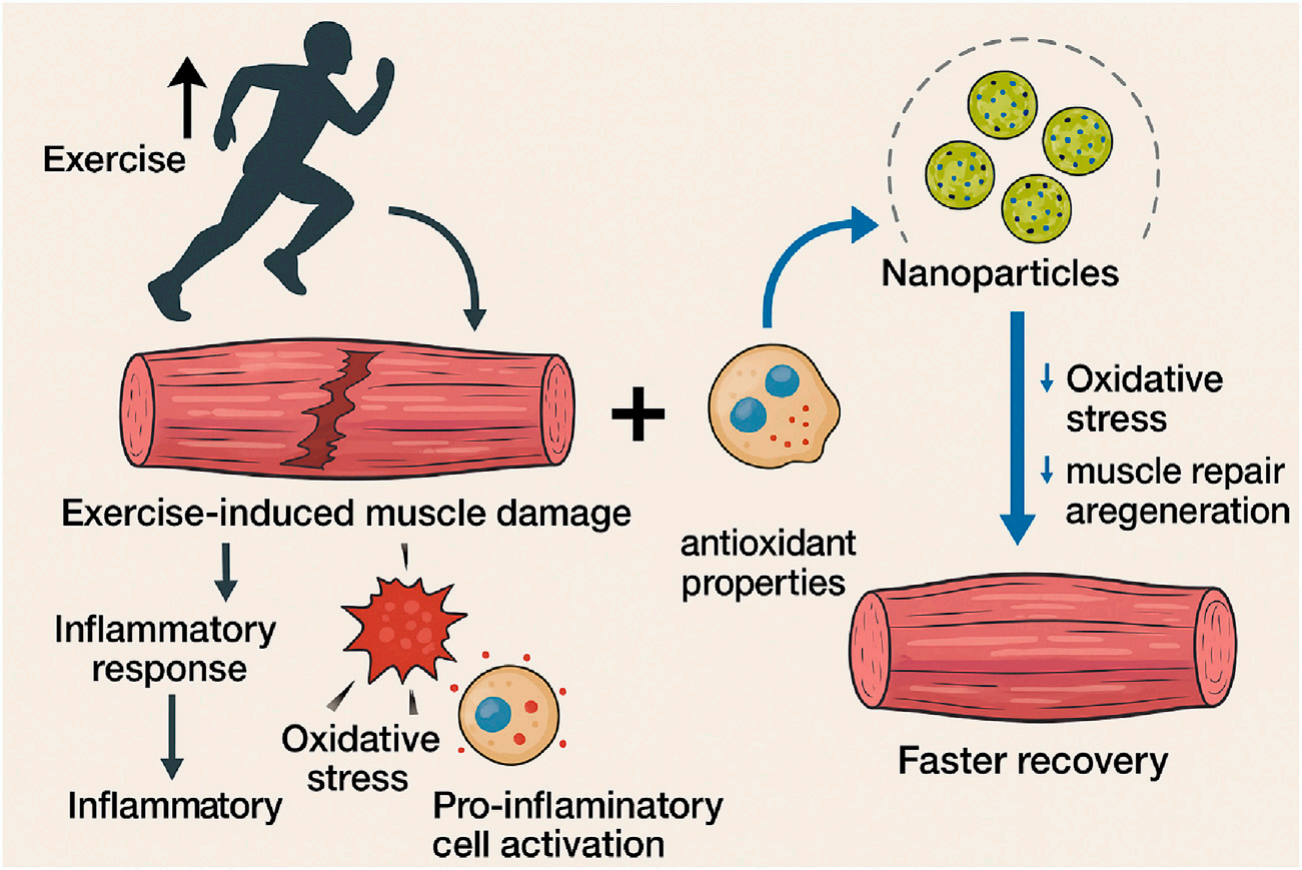
This diagram depicts the mechanism of EIMD and the possible advantages of nanoparticle antioxidants in facilitating muscle recovery. Exercise induces muscle damage, which activates an inflammatory response. This response entails oxidative stress and the activation of pro-inflammatory cells, which ultimately contribute to inflammation. Antioxidant properties are exhibited by introduced nanoparticles. These nanoparticles reduce oxidative stress and facilitate muscle repair and regeneration, leading to accelerated recovery.

Exercise-induced muscle damage (EIMD) and the corresponding inflammatory response are established phenomena that can impede athletic performance and recovery. Intense physical activity results in microtears of muscle fibres, prompting an inflammatory response to repair the damaged tissue (Cai et al., 2025; Roux et al., 2023). This process is crucial for muscle growth and adaptation; however, if not managed appropriately, it may lead to excessive oxidative stress, cellular damage, and delayed recovery (Smith and Miles, 2000; Pyne, 1994). Studies indicate that nanoparticles, especially those exhibiting antioxidant characteristics, can markedly decrease oxidative stress and facilitate quicker recovery after strenuous exercise (Toriumi et al., 2021; Raimondo and Mooney, 2018). The integration of nanoparticles with exercise may enhance the modulation of the immune response, thereby facilitating muscle repair and regeneration (Yang et al., 2022; Awad et al., 2021; Toriumi et al., 2021; Raimondo and Mooney, 2018) (Figure 1).

The connection between exercise and nanoparticles remains a relatively underexplored domain of research. Individual studies examined nanoparticles and exercise’s health benefits; however, the interaction between NPs and exercise remains inadequately understood. Recent studies indicate that nanoparticles could improve physiological responses to exercise, including enhanced endurance, decreased muscle fatigue, and expedited recovery (Yang et al., 2022; Awad et al., 2021; Ziaolhagh et al., 2024; Pinho et al., 2022; Toriumi et al., 2021; Raimondo and Mooney, 2018). The mechanisms governing the interaction between nanoparticles and exercise are not yet fully understood, and the ideal types of nanoparticles and exercise protocols for maximising these synergistic effects are still under investigation.

Moreover, although nanoparticles demonstrate potential in preclinical studies, numerous uncertainties remain concerning their safety, efficacy, and possible side effects when used in conjunction with exercise in human subjects. Concerns regarding the long-term accumulation of nanoparticles in tissues, the immune system’s response to nanoparticle exposure, and the potential adverse effects in vulnerable populations require thorough investigation and resolution (Oostingh et al., 2011; Desai, 2012). This emphasizes the need for more rigorous, human-centered research on safely integrating nanoparticles into exercise regimens for optimal health benefits.

This review seeks to investigate the combined impact of nanoparticles and exercise on health outcomes, addressing existing knowledge gaps. This review provides a thorough examination of existing research, highlighting the potential of nanoparticles to augment exercise benefits and the reciprocal enhancement of nanoparticle-based therapies through exercise. The aim is to analyse the current evidence regarding this novel combination and to pinpoint significant challenges and knowledge gaps that require attention in future research. An enhanced comprehension of the relationship between nanoparticles and exercise may facilitate the creation of innovative therapeutic approaches that integrate exercise and nanotechnology to optimise health outcomes. This is significant in the context of ageing, muscle degeneration, and chronic diseases, as the integration of exercise and nanotechnology may serve as an effective means for enhancing tissue repair, regeneration, and overall wellness. The incorporation of nanoparticles into exercise programs may provide a beneficial approach to enhancing health and wellbeing among diverse age groups and fitness levels.

## Nanoparticles and their role in health

### Different types of nanoparticles

Nanoparticles represent significant advancements in contemporary medicine, providing enhanced functionalities in drug delivery, diagnostics, and therapeutic applications. Microscopic structures, generally 1–100 nm in size, have unique physicochemical properties that make them suitable for biomedical applications. The diminutive dimensions of nanoparticles facilitate their extensive circulation within the body and enable their entry into cells, allowing molecular interactions with biological systems. This capability presents novel opportunities in medical treatment and diagnosis.

### Carbon-based nanoparticles

Carbon-based nanomaterials represent a diverse class of nanostructures with exceptional properties, making them suitable for numerous biomedical applications. These materials have garnered significant attention due to their unique structures, adaptability, and biocompatibility (Gupta et al., 2019; Hosseini et al., 2023).

Carbon nanotubes (CNTs), are cylindrical structures that are originated from rolled graphene sheets. They are among the carbon nanomaterials that have been the subject of the most extensive research in the field of medicine. Because of their porous architecture, high surface-area-to-volume ratio, and high electrical conductivity, they are particularly advantageous for the purposes of drug delivery and diagnostics. CNTs can penetrate cell membranes effectively, enabling the delivery of various therapeutic agents, like drugs, nucleic acids, and vaccines to previously inaccessible destinations (Shabnum et al., 2025). The surface of CNTs can be readily functionalized, which has facilitated their development as gene delivery vectors for conditions such as cancer. These vectors can carry plasmid DNA, miRNA, and siRNA to target sites. Additionally, CNTs’ inherent electrical conductivity makes them excellent candidates for interfacing with electrically excitable tissues, particularly in brain interface investigations (Shabnum et al., 2025). Biomedical applications for graphene, fullerene, and carbon nanotubes are possible. The material known as graphene, which is made up of a single layer of carbon atoms arranged in a honeycomb pattern in two dimensions, possesses remarkable mechanical strength, thermal conductivity, and electrical properties. Fullerenes, which are spherical molecules that are made up entirely of carbon atoms, have a set of physicochemical properties that are one of a kind and can be utilized for the delivery of drugs and therapeutic interventions (Shahazi et al., 2023).

Carbon-based nanomaterials have demonstrated remarkable utility across various medical applications. In diagnostics, they serve as imaging agents, biosensors, and platforms for disease detection and monitoring (Shahazi et al., 2023). Their contrast properties improve tissue visualization and early disease detection (Burlec et al., 2023).

Fullerenes are spherical molecules composed of up to 1500 carbon atoms interconnected via sp2 hybridization (Nasrollahzadeh et al., 2019). Research demonstrates that fullerenes function as carriers for antiviral and antibiotics agents, and used to develop X-ray imaging contrast agents, high-performance MRI (Magnetic Resonance Imaging) contrast agents, gene delivery systems, and photodynamic therapy (Gaur et al., 2021; Lu et al., 2010).

Carbon allotrope graphene has a hexagonal carbon atom arrangement in two dimensions (Geim, 2009).

Carbon nanotubes are cylindrical structures made of carbon, with a diameter of 1 nm and a length varying from 1 to 100 nm. The capacity to penetrate cells through endocytosis or direct membrane insertion, along with their dimensions and morphology, makes them efficient carriers for active substances (Behzadi et al., 2017). Carbon nanofibers can be formed from graphene sheets that are configured in conical or cup-like shapes, rather than the conventional cylindrical form (Yadav et al., 2020). Carbon black is a type of amorphous carbon material that has a diameter that ranges from 20 to 70 nm and is typically spherical in shape. The interaction between the particles is strong enough to cause aggregation, which leads to the formation of agglomerates that are approximately 500 nm in size (Fan et al., 2020).

### Metal-based nanoparticles

Due to their unique properties and medical potential, metal-based NPs, especially those made from noble metals like gold and silver, have garnered scientific attention.

Gold nanoparticles (Au NPs) demonstrate significant biocompatibility and distinctive optical properties, positioning them as suitable candidates for various medical applications. Synthesis can be achieved with precise control over shape and size, and surface modifications can be implemented for specific targeting capabilities. Gold nanoparticles exhibit significant light absorption and scattering properties, rendering them useful for imaging and photothermal therapy in cancer treatment (Kowalska et al., 2024).

One challenge with Au NPs is their tendency to agglomerate, which can destabilize them and diminish their beneficial properties. To address this issue, researchers have developed core-shell nanostructures, such as Au@SiO2, where the gold nanoparticle core is covered with a silica shell. This not only stabilizes the nanoparticles but also allows for further modification and functionalization, expanding their potential applications in medicine (Kowalska et al., 2024).

Both top-down and bottom-up approaches that involve metals can be utilized in the synthesis of metal NPs (Khan MU. et al., 2023). Nanoparticles can be produced from almost all metals through a synthesis process (Salavati-Niasari et al., 2008). The metals most commonly utilised for nanoparticle synthesis include silver (Ag), gold (Au), aluminium (Al), iron (Fe), cobalt (Co), cadmium (Cd), zinc (Zn), and copper (Cu) (Ealia and Saravanakumar, 2017). The size, surface-to-volume ratio, pore size, surface density, electronic surface charge, spherical, crystalline, or amorphous structure, or cylindrical shape, and color are some of the general characteristics that are shared by all nanoparticles. Additionally, nanoparticles are reactive and sensitive to external factors such as humidity, temperature, and light.

Metal oxide NPs are produced through the alteration of the features of the respective metal. FeNPs spontaneously oxidize to iron oxide when exposed to oxygen at room temperature, resulting in enhanced reactivity relative to FeNPs. Metal oxide nanoparticles are primarily produced through physical or chemical synthesis methods. This is due to the fact that they are able to decrease in size when subjected to physical forces, such as those that are present in a rotating reactor, or due to their increased chemical reactivity (Tai et al., 2007). Fe2O3, SiO2, Al2O3, Fe3O4, and TiO2 NPs are typically synthesised using this method. These particles exhibit enhanced properties relative to the metals from which they are derived (Ealia and Saravanakumar, 2017; Vargas-Ortiz et al., 2022).

Quantum dots are nanoscale particles distinguished by their diminutive size and distinctive optical properties. These structures are composed of semiconductor materials, typically lead sulfide (PbS), cadmium telluride (CdTe), or cadmium selenide (CdSe), with diameters between 2 and 10 nm. These materials are employed in many applications, like solar cells, displays, and bioimaging (Le et al., 2022).

The integration of metal nanoparticles with various nanostructures shows considerable promise for targeted medical applications. The amalgamation of gold nanoparticles and silica nanoshells exemplifies the advancement of photothermal agents. These particles preferentially aggregate in tumor tissues and, upon exposure to near-infrared light, generate localized heat that efficiently and specifically eradicates cancer cells. This method, referred to as photothermal therapy, holds promise for cancer treatment (Vines et al., 2019). Creating versatile drug delivery systems can be accomplished by encapsulating gold nanoparticles in liposomes. The precise release of therapeutic agents is made possible by hybrid nanostructures, which also facilitate the delivery of therapeutic agents to specific disease locations, such as tumors. This method enhances the efficacy of chemotherapy while simultaneously reducing the adverse effects that are associated with it (Amini et al., 2023; Koga et al., 2021). Additionally, Mesoporous silica-coated gold nanorods are a theranostic nanoplatform for conventional radiation diagnosis and treatment (Koosha et al., 2021). Hydrogel matrices with silver nanoparticles make antimicrobial wound dressings. These dressings slowly release silver ions, which prevent bacterial infections and speed wound healing (Pangli et al., 2021).

**FIGURE 2:**
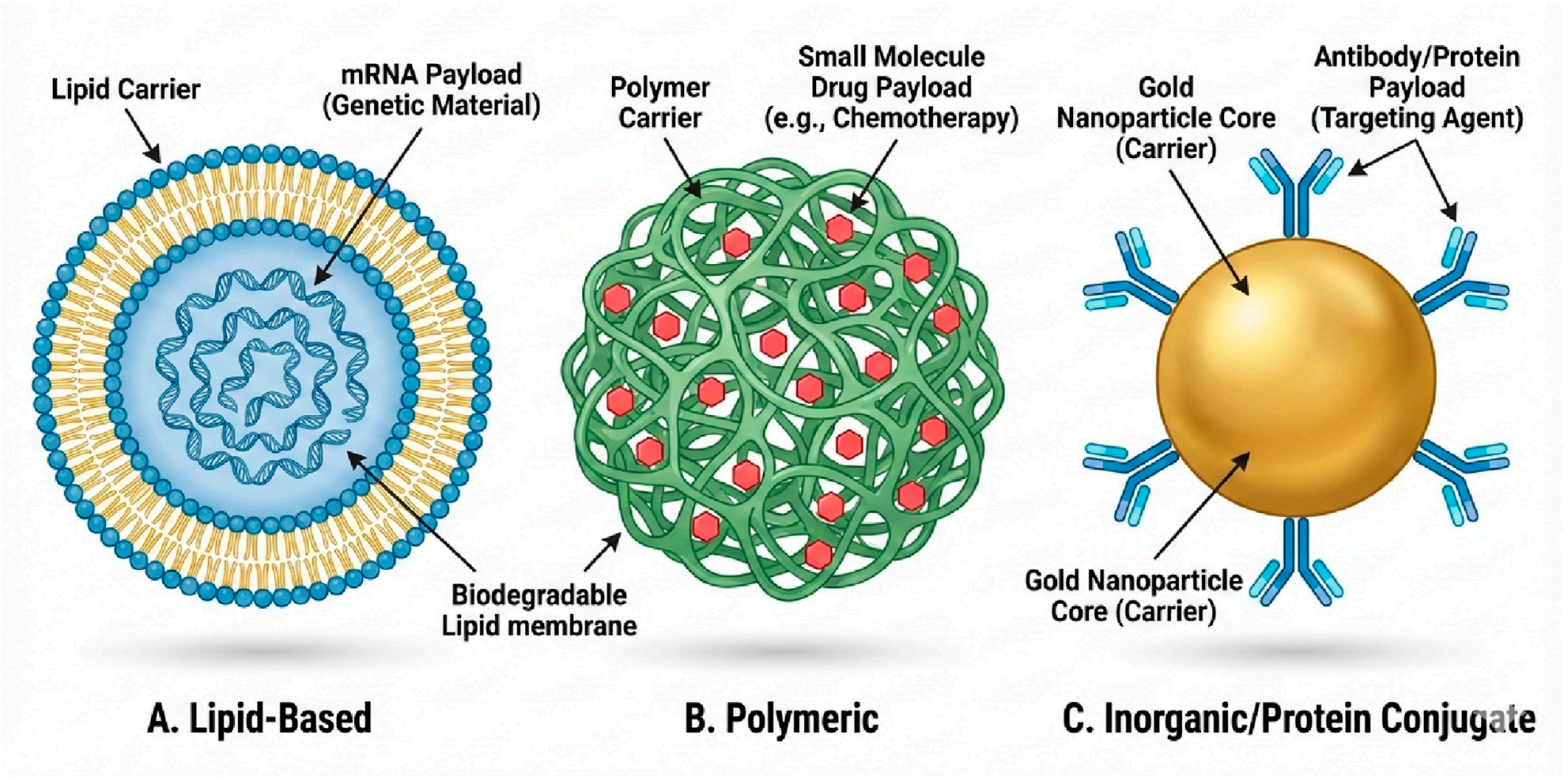
Classification of Therapeutic Nanoparticles by Payload and Clinical Application. **(A)** Lipid-Based Nanoparticles (Liposomes/LNPs): Vesicular carriers capable of encapsulating delicate genetic material. They are currently the leading platform for clinical gene therapies, including mRNA vaccines (e.g., COVID-19) and gene-editing tools (e.g., CRISPR/Cas9) for treating neuromuscular disorders. **(B)** Polymeric Nanoparticles: Biodegradable matrices designed to entrap small-molecule hydrophobic drugs (e.g., chemotherapy agents like paclitaxel). These systems provide controlled, sustained release of the bioactive payload to maintain therapeutic levels over time. **(C)** Inorganic/Conjugate Nanoparticles (e.g., Gold): Solid core carriers functionalized with surface proteins or antibodies. These are primarily used for targeted imaging, photothermal therapy, or the precise delivery of biologic agents to specific cell surface receptors.

Ag NPs are recognised for their significant antimicrobial properties, rendering them effective agents in combating infections (Burlec et al., 2023; Kowalska et al., 2024). Similar to gold nanoparticles, silver nanoparticles can be coated with materials like silica to create core-shell structures (Ag@SiO2), which improve their stability and allow for further functionalization (Burlec et al., 2023) (Figure 2).

### Other inorganic nanoparticles

The nanoparticles that are based on silica belong to a category of inorganic nanomaterials that are made up of silicon and oxygen atoms that are arranged in a crystalline structure. Due to their biocompatibility, chemical and thermal stability, and low toxicity, these materials are suitable for use in a variety of biomedical applications, including imaging, biosensing, and drug delivery (Bruckmann et al., 2022).

In the larger category of inorganic nanoparticles, semimetal nanoparticles are a noteworthy subgroup that can be found within the larger category. The electronic and optical properties of these nanomaterials are particularly noteworthy, and they also have the potential to be used in therapeutic applications. Prominent instances like selenium (Shahmoradi et al., 2022), antimony (Chen et al., 2022) and bismuth nanomaterials, with many biomedical uses (Shahbazi et al., 2020).

### Organic nanoparticles

Lipid-based NPs constitute a crucial class of nanomaterials, demonstrating substantial applications in drug delivery and various medical interventions. These nanoparticles utilise the biocompatibility of lipids to establish efficient delivery systems.

Lipid-based NPs are categorized into two primary types: solid lipid and liposomes NPs (Yetisgin et al., 2020). Liposomes are vesicular entities formed by lipid bilayers that encapsulate an aqueous core. Crucially, they represent the most clinically advanced nanoparticle system, serving as versatile carriers for a wide range of molecular payloads including chemotherapeutic drugs (e.g., Doxorubicin), nucleic acids (DNA, mRNA, siRNA), and proteins. For instance, lipid nanoparticles (LNPs) are the foundational technology for COVID-19 mRNA vaccines, demonstrating their capability to deliver genetic material effectively (Hou et al., 2021). In the context of exercise and myopathy, liposomes can be engineered to deliver anti-inflammatory drugs or gene-editing tools directly to inflamed muscle tissue (Yetisgin et al., 2020; Xu et al., 2022).

Lipid-based systems present several advantages, such as straightforward preparation, high biocompatibility, non-toxicity, and scalability (Xu et al., 2022). Their lipid composition enables effective interaction with cell membranes, thereby facilitating the cellular uptake of therapeutic agents.

Micelles and liposomes are biodegradable nanoparticles characterised by a hollow core region, referred to as a nanocapsule (Esakkimuthu et al., 2014; Khan et al., 2022). These characteristics render them suitable candidates for drug matrices. Micelles serve as the predominant release systems for therapeutic agents that are water-insoluble. The hydrophilic exterior safeguards against physiological processes, while the hydrophobic core houses water-insoluble chemicals, thereby serving as adaptable delivery systems for active compounds (Ahmad et al., 2014).

**FIGURE 3:**
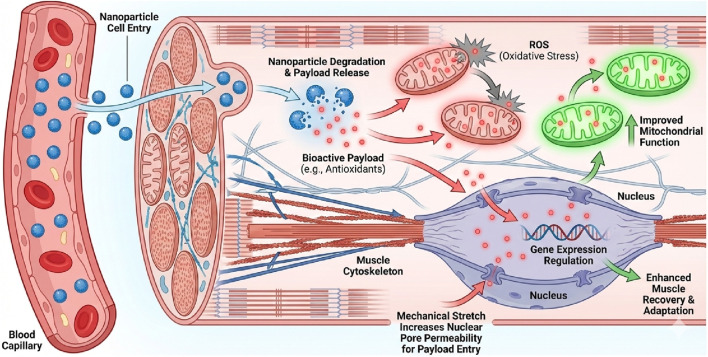
Synergistic Mechanisms of Nanoparticle-Mediated Payload Delivery in Exercising Muscle. The diagram illustrates how exercise creates a receptive physiological environment for nanotherapy (Peña et al., 2022). Delivery and Release: Nanoparticles enter the muscle tissue via enhanced capillary blood flow. Once internalized, the carrier degrades, releasing its bioactive payload (e.g., antioxidants, anti-inflammatory drugs) into the cytoplasm (Koksharov et al., 2022). Mitochondrial Protection: The released payload targets dysfunctional mitochondria, neutralizing Reactive Oxygen Species (ROS) and enhancing biogenesis, distinct from the carrier material itself (Musielak et al., 2022). Nuclear Mechanotransduction: Mechanical forces generated during exercise—specifically stretching—transmit tension through the cytoskeleton to the nucleus via the LINC complex. This mechanical stress transiently widens nuclear pores, facilitating the nuclear translocation of therapeutic payloads (e.g., gene therapies) that would otherwise be excluded, thereby enhancing treatment efficacy for genetic neuromuscular diseases.

Liposomes are vesicles that are synthesized and exhibit a high degree of flexibility. They are also capable of being conjugated with a variety of lipid substances, which allows them to have a considerable range of sizes. The principal advantage of liposomes is their ability to merge with cell membranes, resulting in the release of the active ingredient (Daraee et al., 2016; Buse and El-Aneed, 2010). Multilamellar liposomes comprise several lipid layers interspersed with hydrophilic layers, making them efficient for encapsulating both lipophilic and hydrophilic compounds (Oberholzer and Luisi, 2002). Currently, liposomes are extensively studied for various therapeutic applications, like antimicrobial therapy, cancer diagnosis and treatment, and targeted drug delivery (Daraee et al., 2016) (Figure 3).

Dendrimers are branched polymeric structures characterised by a tree-like morphology and precise molecular architecture. Their distinctive structure offers various surface functional groups that can be altered for targeted applications or drug attachment. Polyamidoamine (PAMAM) dendrimers is utilised for the delivery of N-acetyl-cysteine, illustrating their potential in targeted drug delivery. Micelles are self-assembled structures created by amphiphilic polymers in aqueous environments. Their structure generally comprises a hydrophobic core capable of encapsulating poorly water-soluble drugs, alongside a hydrophilic shell that maintains stability during circulation. Both polypropylene sulfide-PEG-serine-folic acid zinc phthalocyanine micelles containing doxorubicin and polypropylene sulfide-polyaspartate polymeric micelles encapsulating paclitaxel are examples of such micelles (Yetisgin et al., 2020).

Dendrimers are widely utilised in clinical applications owing to their small size (1–5 nm) and structural properties, which facilitate coupling with biocompatible compounds to reduce cytotoxicity. They serve as delivery systems for vaccines, genes, or pharmaceuticals (Palmerston et al., 2017; Hsu et al., 2017). Despite their versatility, certain compounds exhibit a significant risk of aggregation and toxicity, with only a limited number receiving FDA (Food and Drug Administration) approval (Mitchell et al., 2021).

Nanogels are cross-linked polymeric networks that swell in water. Nanogels can encapsulate multiple therapeutic molecules and reduce drug leakage compared to other nano-carrier systems. They are administered parentally or mucosally and used in biosensors, biochemical separation, drug delivery, and antitumor therapy (Yetisgin et al., 2020).

Protein and drug conjugates are other polymer-based nanoparticles with unique medical benefits. The variety of polymeric materials allows nanoparticles with tailored properties to address therapeutic challenges (Yetisgin et al., 2020).

Synthetic or natural polymeric nanocapsules or nanospheres. Therapeutic agents can be concentrated in synthetic NPs for targeted release (Vardaxi et al., 2022; Spirescu et al., 2021). Several studies examined PMMA dispersions’ production and use (Shabanova et al., 2016; Nemtsev et al., 2019). PMMA, a common amorphous synthetic polymer, might be useful in biomedicine due to its low cost, low toxicity, minimal tissue inflammation, and easy processability (Batista et al., 2020).

Protein polymers are isolated proteins from animals or plants like gelatin, collagen, albumin, or elastin that self-assemble into protein nanoparticles. Genetically engineered protein subunits self-assemble into effective drug delivery vehicles using polymer-based nanoparticles (Kianfar, 2021; Aljabali et al., 2022). Abraxane® is an FDA-approved albumin-bound nanoparticle formulation of paclitaxel, which enhances the delivery of the hydrophobic chemotherapy drug to tumor tissues while eliminating the need for toxic solvents. This platform demonstrates how protein-based nanocarriers can improve the therapeutic index of toxic drugs (Herrera Estrada and Champion, 2015). Ontak is a protein that has been engineered to combine IL-2 with a nanoparticle formulation of diphtheria toxin. It is utilized in the treatment of cutaneous T-cell lymphoma that is either persistent or recurrent (Hong et al., 2020).

The International Union of Pure and Applied Chemistry defines nanogels as gel particles of any shape with an equivalent diameter of 1–100 nm (Alemán et al., 2007). In comparison to alternative nanocarrier systems, nanogels present several advantages, such as a diminished rate of premature drug release, the ability to encapsulate therapeutic agents within a singular formulation, and the convenience of administration through parenteral or mucosal routes. Their applications have been extensively researched, and they include the delivery of nucleic acids, cytokines, and vaccines, particularly nasal vaccines, which have shown a great deal of promise (Neamtu et al., 2017). Table 1 provides a summary of different categories of NPs.

**TABLE 1:** Different types of nanoparticles and their application in medicine.

Nanoparticle type	Properties	Key payloads (DNA, RNA, drugs, proteins)	Clinical/Disease applications	References
Carbon-Based				
CNTs	High surface area, conductive	DNA, siRNA, proteins	Gene therapy, neural interfaces, cancer targeting	Gao et al. (2024), Alfei et al. (2025), Singh and Kumar (2022)
Metal-Based				
Au NPs	Biocompatible, optical properties	Antibodies, chemotherapy drugs	Photothermal therapy, diagnostic imaging	Vashitha and Khan (2025); Ama et al. (2025), Koç et al. (2025)
Organic				
`	Bilayer vesicle, biocompatible	mRNA, siRNA, Doxorubicin	COVID-19 vaccines, Cancer chemotherapy, Duchenne Muscular Dystrophy (gene delivery)	Moazzam et al. (2024), Khan et al. (2023b)
Solid Lipid NPs	Solid lipid core, stable	Lipophilic antioxidants (Resveratrol)	Nutraceutical delivery, fatigue reduction, metabolic disorders	Cruz et al. (2025), Pandita et al. (2014)
Dendrimers	Branched, precise architecture	DNA, N-acetyl-cysteine	Gene delivery, targeted anti-inflammatory therapy	Stachura et al. (2024), Fadaei et al. (2025)
Nanogels	Cross-linked, swelling	Vaccines, Cytokines	Nasal vaccines, immunotherapy	Saleh et al. (2025), Zhang et al. (2025), Pinelli et al. (2023)

## An overview of exercise and its effects on the body

**FIGURE 4:**
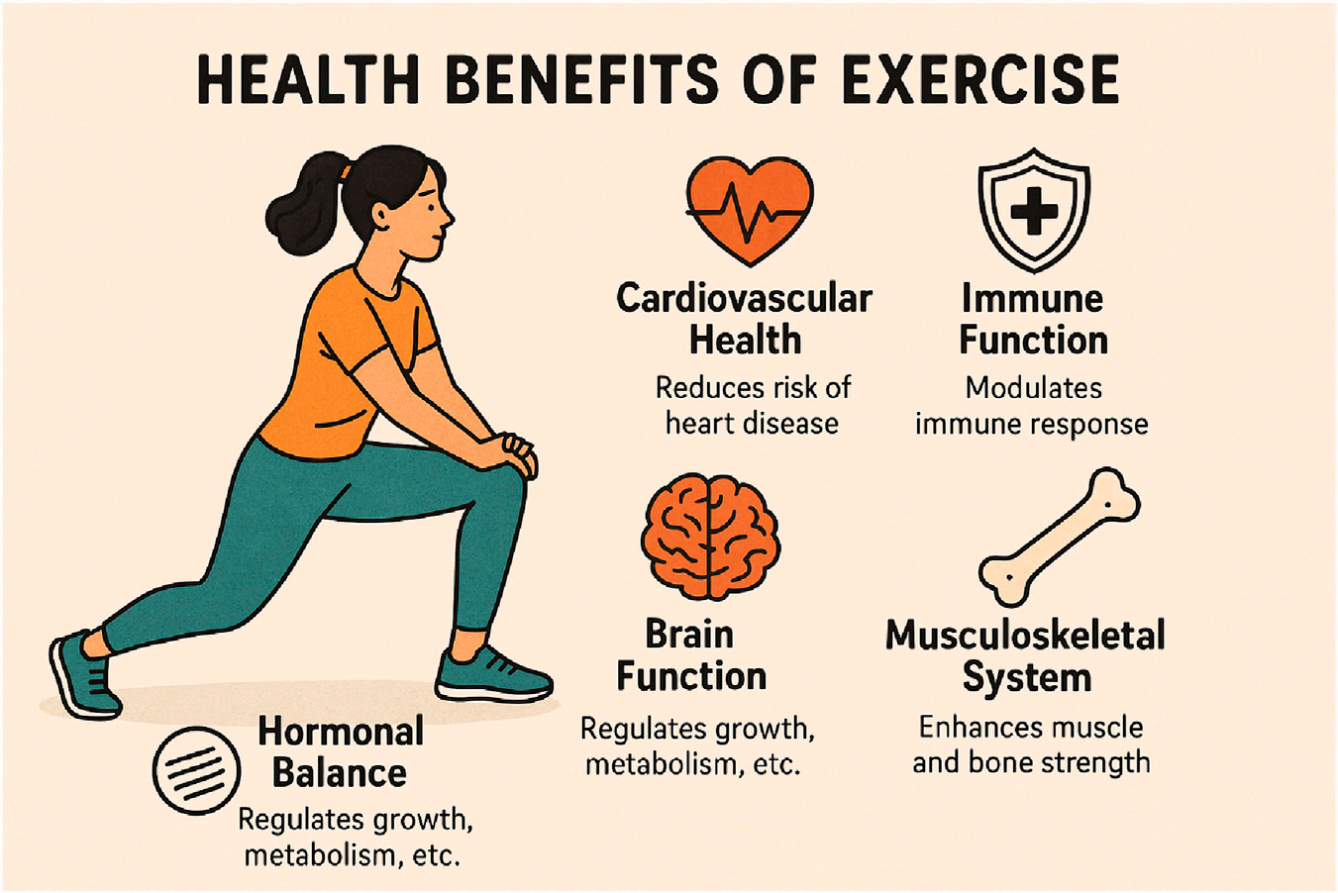
This infographic illustrates the broad health benefits of regular exercise, covering cardiovascular health, brain function, hormonal balance, immune function, and musculoskeletal strength. Exercise improves heart function, reduces inflammation, and helps prevent cardiovascular diseases. It boosts cognitive performance, enhances neuroplasticity, and protects against cognitive decline, especially in older adults. Exercise also supports healthy hormonal levels, crucial for metabolism and tissue repair. Additionally, it modulates the immune system by lowering inflammation and aiding in recovery. Lastly, physical activity strengthens muscles, improves flexibility, and increases bone density, ensuring overall musculoskeletal integrity.

Physical exercise has emerged as a powerful intervention with profound effects across multiple body systems (Figure 4). Recent research demonstrates that regular physical activity delivers significant benefits to cardiovascular health, brain function, hormonal balance, immune response, and musculoskeletal integrity. These effects extend beyond mere physical fitness, contributing to disease prevention, cognitive enhancement, and improved quality of life across different age groups. However, the specific effects vary based on exercise type, intensity, duration, and timing, with potential risks when performed excessively or improperly.

Exercise is essential for primary and secondary cardiovascular diseases (CVD) prevention. The pandemic of physical inactivity closely parallels the widespread prevalence of CVDs, establishing a clear connection between sedentary behavior and cardiovascular health. Exercise induces several beneficial adaptations in the cardiovascular system, including a healthier metabolic profile and attenuation of systemic chronic inflammation, which are key factors in cardiovascular disease development. At the vascular level, regular exercise exerts antiatherogenic effects, helping to prevent the buildup of plaque in arteries and improving endothelial function. Additionally, exercise promotes myocardial regeneration and cardioprotection at the heart tissue level, enhancing the heart’s resilience and functional capacity (Valenzuela et al., 2023).

The timing of exercise may influence its cardiovascular benefits. Recent findings indicate that evening exercise may offer superior cardiovascular benefits regarding blood pressure and autonomic control when compared to morning exercise. This chronobiological aspect of exercise highlights how our body’s circadian rhythms interact with physical activity to optimize physiological responses 9. Furthermore, exercise has the potential to realign the circadian system, thereby mitigating disruptions caused by contemporary lifestyle elements, including nighttime exposure to artificial light, which is linked to heightened cardiovascular risk. The combined evidence indicates that exercise serves as a potent intervention for cardiovascular health through multiple mechanistic pathways involving metabolic, inflammatory, vascular, and myocardial adaptations (Brito et al., 2022).

Exercise demonstrates remarkable effects on brain function and cognitive performance across the lifespan. Regular physical activity enhances cognitive abilities through multiple mechanisms, with particularly strong evidence for its protective effects against cognitive decline and dementia in older adults (Hillman et al., 2008; Yamasaki, 2023). Aerobic physical activity has emerged as especially beneficial for maintaining cognitive function, with open-skill exercises (those requiring adaptation to changing environments, like tennis or dance) showing stronger protective effects than closed-skill exercises (repetitive activities in stable environments, like swimming or running) 10. These cognitive benefits stem from exercise’s ability to improve cerebral blood flow, enhance neuroplasticity, and reduce neuroinflammation (Yamasaki, 2023).

Recent research on Brain Endurance Training (BET) provides intriguing evidence of how combined physical and cognitive training enhances performance. BET, which involves performing cognitive tasks during or around physical training, has been shown to improve not only physical performance but also cognitive function beyond what physical training alone achieves. In one study, participants who underwent BET showed significant improvements in various calisthenic exercises and demonstrated evidence of near transfer of training to novel exercise and cognitive tasks (Dallaway et al., 2024). This suggests that exercise’s effects on the brain are not limited to structural changes but also include functional adaptations that can be strategically enhanced through combined interventions targeting both physical and cognitive domains simultaneously.

The preventive effects of exercise against cognitive decline become increasingly important in aging populations. No curative treatment for dementia, including Alzheimer’s disease, exists, so optimal non-pharmaceutical preventive interventions are essential. Physical inactivity is a modifiable risk factor for dementia, especially AD (Yamasaki, 2023). The accessibility and relatively low cost of physical activity interventions make exercise an attractive and practical approach to maintaining cognitive health throughout the aging process, offering a powerful complement or alternative to pharmaceutical approaches.

Exercise induces significant changes in hormonal status, with particularly notable effects on GH, DHEA-S, and estrogen levels. These hormonal adaptations play crucial roles in metabolism, tissue repair, and overall physiological function. In elderly women, a 12-week combined exercise program of Korean dance and yoga significantly increased levels of growth hormone, DHEA-S, and estrogen compared to control groups. These hormonal improvements occurred alongside enhancements in balance, flexibility, and muscle strength, demonstrating the interconnected nature of hormonal and physical adaptations to exercise (Im et al., 2019). The hormonal benefits of exercise are particularly valuable for postmenopausal women, who typically experience age-associated hormonal declines that can contribute to various health issues.

Resistance training appears to be especially effective in combating age-related hormonal changes. Improvements in aging-related hormones such as growth hormone, estradiol, DHEA-S, and IGF-1were observed in postmenopausal women with stage 1 hypertension who participated in a resistance band exercise training program for a period of 12 weeks. These hormonal improvements were accompanied by beneficial changes in blood pressure and body composition, highlighting how exercise-induced hormonal changes contribute to broader health improvements (Son et al., 2020). The relationship between exercise and hormones is bidirectional–exercise influences hormone levels, while hormones affect exercise capacity and adaptations.

However, excessive exercise without adequate recovery can disrupt hormonal balance in a negative way. Overtraining can lead to decreased testosterone levels and elevated cortisol levels. This hormonal dysregulation can contribute to numerous negative outcomes, including increased fatigue, reduced performance, and compromised recovery (Bereda, 2022). The relationship between exercise and hormonal status follows an inverted U-shaped curve, where appropriate levels of activity optimize hormonal function, while excessive exercise can lead to maladaptive hormonal responses that undermine health and performance.

**FIGURE 5:**
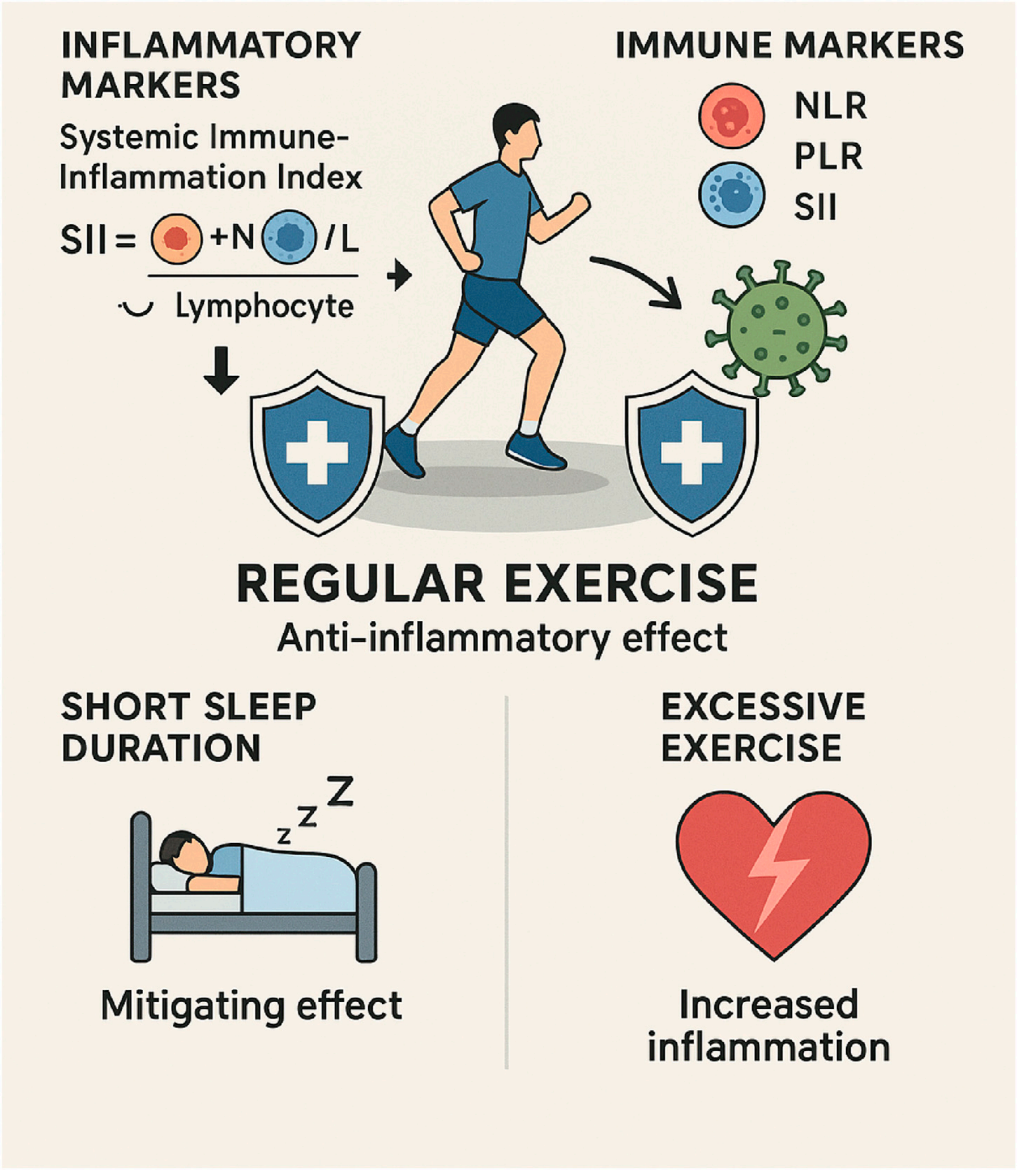
This image depicts the positive effects that exercise has on immune function. The findings demonstrate that engaging in regular physical activity can reduce inflammation, modulate immune markers, and improve the quality of sleep. Inflammatory markers, such as the SII, can be reduced through the presence of exercise, which also helps to maintain a balanced immune response. As an additional benefit, it reduces the adverse effects of insufficient sleep, which has been linked to increased inflammation. In addition, the infographic highlights the potential dangers of engaging in excessive physical activity, which can interfere with immune function and make one more susceptible to illness. Generally speaking, physical activity helps improve the effectiveness of the immune system and reduces chronic inflammation.

Regular physical activity significantly influences the immune system, especially regarding inflammatory markers that indicate systemic inflammation. The Systemic Immune-Inflammation Index (SII), calculated as platelet and neutrophil counts divided by lymphocyte counts, indicates inflammatory potential and is increasingly used to assess exercise-induced immunological changes (Walzik et al., 2021). Research indicates that physical exercise shows significant negative associations with SII levels, suggesting an anti-inflammatory effect of regular activity. This relationship appears to have a saturation point, with research suggesting that exercising up to 2400 MET-minutes/week (a measure of exercise volume) is associated with lower SII levels, after which additional benefits diminish (You et al., 2024) (Figure 5).

The relationship between exercise and inflammation is particularly interesting in populations with compromised sleep duration. Short sleep duration is associated with increased inflammation, but regular exercise demonstrates a mitigating effect on this relationship. This finding highlights the potential of exercise as an intervention to counteract the inflammatory consequences of poor sleep, which is increasingly common in modern society (You et al., 2024). Exercise reduces pro-inflammatory cytokines, modulates immune cell function and distribution, and increases anti-inflammatory cytokines (Walzik et al., 2021).

Exercise-induced changes in integrative inflammation markers like the Neutrophil-to-Lymphocyte Ratio (NLR), Platelet-to-Lymphocyte Ratio (PLR), and SII offer promising avenues for monitoring exercise responses in both health and performance settings. These markers respond to both acute and chronic exercise, suggesting their potential usefulness in indicating exercise strain, recovery status, and instances of overtraining and increased infection risk in athletic populations. In clinical populations experiencing chronic inflammation, these markers can elucidate the anti-inflammatory benefits of consistent exercise regimens (Walzik et al., 2021). The accessibility of these markers, requiring only basic blood tests, enhances their practical utility in various exercise and health contexts.

Despite its numerous benefits, excessive or inappropriate exercise can pose significant risks to physical and psychological health. Overtraining syndrome, characterized by persistent fatigue, performance decrements, and physiological disturbances, represents a serious concern for individuals engaged in intensive training programs. German research in Heart suggests that excessive high-intensity exercise may increase the risk of heart attack or stroke in heart disease patients (Bereda, 2022). This highlights the importance of individualized exercise prescription that considers existing health conditions and appropriate progression of training intensity and volume.

**FIGURE 6:**
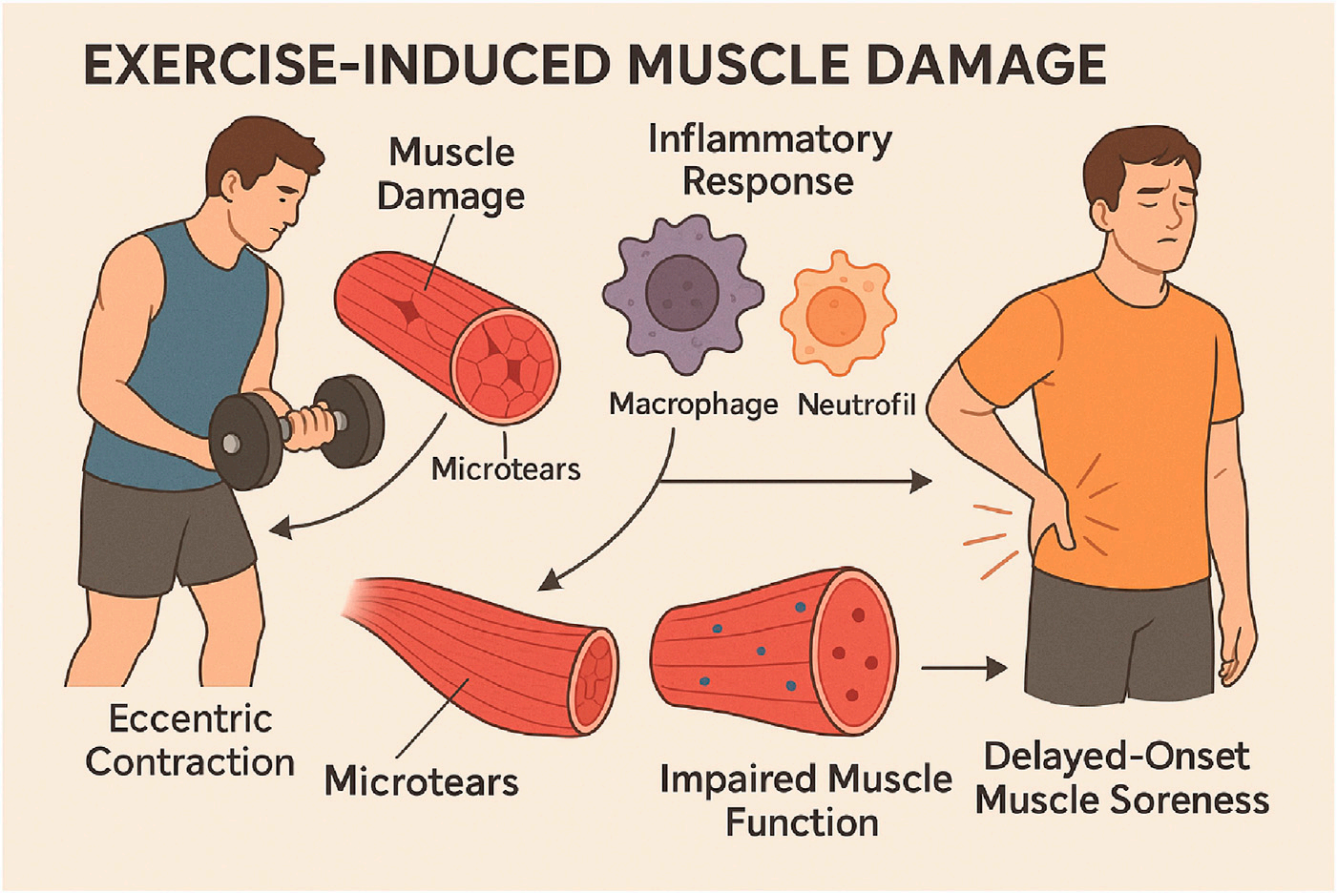
This infographic elucidates EIMD, outlining the stages of muscle damage resulting from intense or unfamiliar exercise. The process begins with initial muscle fibre damage, especially due to eccentric contractions, followed by a pro-inflammatory phase characterised by the release of cytokines like TNF-α and IL-6, which facilitate the recruitment of immune cells for tissue repair. The infographic illustrates the anti-inflammatory phase, during which macrophages remove damaged tissue and activate satellite cells to facilitate muscle regeneration. This also demonstrates DOMS, which peaks 24–72 h after exercise, and highlights the significance of biomarkers such as CK and LDH in assessing muscle damage and recovery.

Exercise-induced muscle damage (EIMD) refers to structural and functional disruptions in muscle fibers caused by intense or unaccustomed physical activity, particularly eccentric exercises (Tee et al., 2007; Owens et al., 2019). This damage is characterized by microtears in muscle fibers, especially within sarcomeres, due to high mechanical tension during eccentric contractions like downhill running or lowering weights. The inflammatory response following EIMD is a critical component of tissue repair and adaptation. It begins with a pro-inflammatory phase where cytokines like IL-6 and TNF-α are released, recruiting macrophages and neutrophils to the site of injury (Pyne, 1994; Markus et al., 2021; Fatouros and Jamurtas, 2016). This is followed by an anti-inflammatory phase, where macrophages clear debris and activate satellite cells for muscle repair. Biomarkers of EIMD include elevated levels of CK, LDH, and inflammatory cytokines like IL-6 and IL-8 (Markus et al., 2021; Santos et al., 2020; Romero-Parra et al., 2021). EIMD can impair muscle function, reduce strength, and cause Delayed-Onset Muscle Soreness (DOMS), peaking 24–72 h post-exercise (Figure 4) (Tanabe et al., 2021) (Figure 6).

## Synergistic effects: nanoparticles and exercise

Preclinical studies have begun to explore the synergistic potential of combining specific nanoparticle formulations with exercise protocols. These investigations, primarily in rodent models, provide initial evidence that nanoparticle-based interventions can enhance performance, reduce exercise-induced damage, and improve physiological markers more effectively than either exercise or the active compound alone. The following studies exemplify the current research in this emerging field.

A study by Toriumi et al. examined the effects of an antioxidant redox nanoparticle (RNP), which acts as a self-assembling ROS scavenger, on the running performance of rats. They found that RNP administration increased running time in a dose-dependent manner. In contrast, a standard low-molecular-weight antioxidant decreased performance. After exercise, the RNP-treated group showed significantly less oxidative stress and damage to red blood cells (RBCs) compared to controls, maintaining levels similar to sedentary animals. The authors concluded that the prolonged circulation of RNPs allows for effective scavenging of excess ROS in the bloodstream, thereby protecting RBCs and enhancing endurance performance (Toriumi et al., 2021).

Resveratrol exhibits antioxidant, antitumorigenic, and anti-inflammatory properties (Ramírez-Garza et al., 2018). Resveratrol may be useful in preventing fatigue and improving exercise performance, among other potential benefits (Dolinsky et al., 2012). The clinical application of resveratrol remains challenging due to its low solubility and bioavailability (Salehi et al., 2018; Qin et al., 2020). With this in mind, self-nanoemulsifying and solid lipid nanoparticles the drug delivery system have been utilised in order to circumvent these limitations and broaden the scope of applications for resveratrol utilization (Yen et al., 2017; Sun et al., 2019).

During the course of an 8-week running training regimen, C57BL/6J mice were evaluated. The training began at a speed of 10 m per minute for 120 min per day, and then the speed was gradually increased to 20 m per minute until the mice were exhausted (Sun et al., 2019). Supplements, which consisted of resveratrol-loaded solid lipid nanoparticles, free-form resveratrol, or a control group, were given to the participants 1 hour before they began their workouts, 6 days a week, for a total of 8 weeks. When compared to the control group, mice that consumed resveratrol-loaded solid lipid NPs before training experienced a 28.7% increase in the average running distance to exhaustion (8617.8 m) compared to the control group (6695.3 m). Despite this, there was no discernible difference between it and mice that were given free-form resveratrol (7364.5 m). Subsequent to the consumption of resveratrol-loaded solid lipid NPs, the respiratory exchange ratio experienced a decrease during exercise of low to moderate intensity. The researchers came to the conclusion that the consumption of resveratrol-loaded solid lipid nanoparticles, which increased the rate of fat oxidation, was associated with a decrease in the respiratory exchange ratio, which is indicative of a substrate shift towards fat metabolism. The fact that the data on respiratory exchange ratio were only presented in graphical form is an important point to keep in mind because it restricts the ability to quantify the changes in fat oxidation rate. In order to improve mitochondrial function, resveratrol-loaded solid lipid NPs were able to improve the equilibrium between mitochondrial biogenesis and mitophagy. This allowed for more precise regulation of the production of functional mitochondria and selective removal of dysfunctional mitochondria.

An investigation was conducted to determine how the running performance of C57BL/6J mice was affected by the presence of resveratrol-loaded solid lipid NPs (Qin et al., 2020). Every day of the training regimen consisted of a total of one hundred and 20 minutes of running, and the routine was carried out over the course of a period of 4 weeks. In addition, mice were given either resveratrol-loaded solid lipid nanoparticles, free-form resveratrol, or a placebo once daily, 6 days a week, for a total of 8 weeks, beginning 4 weeks prior to the training. This was done for a total of 8 weeks. In total, this was carried out for a period of 8 weeks. During a test of forced running capacity, the group that was given resveratrol-loaded solid lipid nanoparticles as a supplement had a significantly longer time to exhaustion and running distance than the group that was given a placebo. This was the case when compared to the placebo group. On the other hand, the group that was given free-form resveratrol as a supplement did not experience these effects (the data were only presented in a graphical format). Additionally, resveratrol-loaded solid lipid nanoparticles improved antioxidant defense, decreased oxidative stress and lipid peroxidation, and made a reduction in oxidative stress (the data was only presented graphically). These findings were presented using a graph. It was observed that the group that was supplemented with resveratrol-loaded solid lipid nanoparticles had a higher incidence of fibers that had a normal shape. Furthermore, the group displayed lower levels of inflammatory infiltration, oedema, and myonecrosis than the other groups. In conclusion, the integrity of the muscle fibers was preserved to a greater extent in this respective group. These findings led the authors to the conclusion that solid lipid nanoparticles loaded with resveratrol could potentially be useful in enhancing endurance capacity and facilitating recovery from strenuous exercise. This conclusion was reached on the basis of the findings presented in the previous paragraph.

An investigation that was conducted not too long ago compared the bioavailability of resveratrol that was administered through a self-nanoemulsifying drug delivery system to that of its non-nanoencapsulated manifestation (Yen et al., 2017). It was determined that the pharmacokinetics of the compound could be evaluated by collecting blood samples from Sprague-Dawley rats in a systematic manner. It was found that the mean maximum concentration of resveratrol in blood was 2.2 times higher when it was delivered in nanoparticles as opposed to its free form. The measurements were 869.2 ± 112.2 ng/mL for the nanoparticles and 386.2 ± 68.4 ng/mL for the free form. When compared to its free form, which has a bioavailability of 3.0% ± 0.8%, the oral bioavailability of resveratrol delivered in nanoparticles was found to be 9.5% ± 1.5%, which is three times higher on average. The administration of resveratrol via a self-nanoemulsifying drug delivery system led to a time to exhaustion during an exhaustive swimming test that was 2.1 times greater than the vehicle group and 1.8 times greater than the resveratrol free form group. This was evident when compared to the results of the vehicle group. The group receiving resveratrol via a self-nanoemulsifying drug delivery system exhibited decreased serum ammonia levels and increased clearance of plasma glucose and lactate. This contrasted with the group that received the vehicle.

In an independent study, thirty-five male Wistar rats were randomly assigned to one of five groups of seven. These groups included a control group, a saline group, a ZnO NPs group, an exercise group, and a group that included both exercise and ZnO NPs. 1 mg/kg of ZnO NPs was given to each of the subjects who were assigned to either the ZnO NPs or exercise + ZnO NPs groups. An exercise program that involved treadmills was carried out by rats. Treatments were carried out over the course of a period of 4 weeks, with 5 days included in each week. SOD activity, apelin, MDA, AT1R, and Ang II concentrations in heart tissue were measured after a treatment period of 4 weeks. These measurements were taken after the treatment period. There was a significant increase in the levels of Ang II, MDA, and AT1R in the heart after the administration of ZnO nanoparticles, while there was a significant decrease in the levels of SOD activity and apelin. The levels of Ang II, MDA, and AT1R in the hearts of rats that were exposed to ZnO nanoparticles were significantly reduced, and the levels of SOD activity and apelin were significantly increased as a result of aerobic exercise. The findings suggest that the attenuation of the Ang II-AT1R signaling pathway that is induced by exercise is facilitated by decreased lipid peroxidation, increased antioxidant defense, and enhanced apelin synthesis. This may function as a protective mechanism against cardiac oxidative stress induced by ZnO nanoparticles simultaneously (Habibian et al., 2023).

When it is prepared, green tea, which is also referred to as GT, is an antioxidant that occurs naturally and easily oxidizes its antioxidant properties. The incorporation of bioactive compounds is achieved through the utilization of nanostructured systems that are referred to as LLCs. High-intensity interval training, also referred to as HIIT, is a form of physical activity that increases the proportion of ROS that are generated. As a consequence of this, the objective of this research was to investigate the effects of GT and GT loaded in LLC (Lyotropic Liquid Crystal) on animals that were being subjected to HIIT, taking into consideration their body mass in addition to their haematological, biochemical, and histological parameters. For the purpose of obtaining nanoparticles of LLC (NP-LLC), monoolein, GT in infusion, and Poloxamer 407 were successfully combined. This was done according to the protocol. Three of the six groups that were randomly assigned to healthy male rats were the Control (C) group, the GT group, the GT-NP-LLC group, the Exercise (Ex) group, the GT + Ex group, and the GT-NP-LLC + Ex group. Six rats were included in each of the groups. When compared to the group that served as the control, all of the groups that participated in high-intensity interval training (HIIT) had significantly lower body weights of their own. When compared to the group that served as the control, the percentages of body mass reduction for Ex, GT + Ex, GT-NP-LLC + Ex, and GT-NP-LLC were 11.3%, 13.0%, 10.0%, and 11.0%, respectively. The control group was the majority of the participants. Triglyceride levels were found to be reduced more effectively by GT-NP-LLC and Ex than by C. This was discovered through research. In comparison to HIIT training on its own, supplementation with GT and GT-NP-LLC led to an increase in muscle hypertrophy of 25% and 21%, respectively, as well as an improvement in physical conditioning and a reduction in the accumulation of body weight. In addition, the combination of these two supplements led to a reduction in the accumulation of body weight within the body. It is therefore encouraging that the effects of GT-NP-LLC itself on body mass and biochemical parameters are positive, which suggests that NP-LLC may be able to improve the bioavailability of GT. On top of that, the effects of GT-NP-LLC on body mass are particularly encouraging (Nieri et al., 2022).

The synergistic benefits of combining nanoparticles with exercise appear to operate through several key physiological mechanisms:

Enhanced antioxidant activity and reduced oxidative stress: Nanoencapsulated compounds provided superior protection against exercise-induced oxidative damage compared to conventional formulations (Lima et al., 2024):

- Enhanced antioxidant activity via bioactive payloads: Nanoparticle-encapsulated antioxidants (e.g., resveratrol, curcumin) provide superior protection against exercise-induced oxidative damage compared to free compounds, likely due to improved bioavailability and sustained release (Lima et al., 2024).
- Anti-inflammatory effects mediated by targeted delivery: The efficient transport of anti-inflammatory agents via nanoparticles reduces inflammatory infiltration, edema, and myonecrosis during intensive exercise regimens, effectively ameliorating the inflammatory cascade.
- Maintenance of muscle integrity: Studies report better preservation of normal muscle fiber shape and structure, attributed to the enhanced cytoprotective effects of the delivered compounds rather than the carrier material itself.
- Improved mitochondrial function: Bioactive compounds delivered via nanocarriers enhance mitochondrial quality and function, improving energy production during exercise by optimizing the pharmacokinetics of mitochondrial-targeting agents.
- Enhanced energy substrate availability: Nano-formulations of metabolic regulators have been shown to elevate glucose levels during exercise and increase muscle and hepatic glycogen stores more effectively than standard oral supplements.

The findings indicate that the protective and performance-enhancing effects of nanoparticles may synergistically interact with exercise-induced adaptations, yielding superior outcomes compared to either intervention in isolation. Table 2 provides an overview about the current research on synergistic effects of exercise and nanoparticles.

**TABLE 2:** Current research on synergistic effects of nanoparticles and exercise.

Nanoparticle type	Active compound	Exercise protocol	Observed effects	Mechanisms	References
RNP	Redox Nanoparticle (self-assembling antioxidant)	Running experiment in rats	Prolonged running time, maintained RBC levels, and reduced oxidative stress	Enhancement of exercise performance through ROS removal, suppression of RBC oxidative stress	Toriumi et al. (2021)
Solid Lipid Nanoparticles (SLNs)	Resveratrol	8-week running protocol in C57BL/6J mice	28.7% increased running distance to exhaustion, improved fat oxidation, mitochondrial biogenesis	Resveratrol-loaded SLNs increased fat oxidation and mitochondrial function	Qin et al. (2020)
ZnO NPs	Zinc Oxide	4-week treadmill exercise in Wistar rats	Increased SOD activity, decreased oxidative stress, and improved heart tissue markers	ZnO NPs reduced oxidative stress and improved antioxidant defense during exercise	Habibian et al. (2023)
GT-NP-LLC	Green Tea (GT)	HIIT protocol in rats	Improved muscle hypertrophy, better physical conditioning, reduced body weight gain	GT-NP-LLC improved bioavailability of GT and enhanced exercise effects	Nieri et al. (2022)
CuNP	Copper	Swimming for 90 min, 4 weeks	Reduced myocardial damage, decreased oxidative stress, reduced inflammatory cytokines, increased NO bioavailability	CuNP and exercise training favorably phosphorylate GSK-3Î^2^ pathways, reducing oxidative stress and apoptosis	–
RNPO	Redox Nanoparticle (self-assembling antioxidant)	Running until exhaustion, 80 min	Significantly enhanced running time, protected GI tract from damage, prevented increase in plasma LPS levels, reduced RBC and skeletal muscle damage	Continuous scavenging of excessive intestinal ROS protects gut and improves exercise performance	–
CuNP	Copper	Swimming for 90 min, 4 weeks	Reduced myocardial damage, decreased oxidative stress, reduced inflammatory cytokines, increased NO bioavailability	CuNP and exercise training favorably phosphorylate GSK-3Î^2^ pathways, reducing oxidative stress and apoptosis	Feizolahi et al. (2024)
L-ACN	L-Arginine	Swimming exercise, 5 days/week for 6 weeks	Increased HAND2 and TBX5 mRNA and protein expression, improved cardiac function, and cardiomyocyte signaling in aging rats	L-ACN supplementation combined with swimming exercise strengthens cardiomyocyte signaling via HAND2 and TBX5, offering cardioprotective potential	Zargani et al. (2022)

At the cellular level, exercise creates a physiologically receptive environment that may enhance nanoparticle delivery and efficacy. Physical activity increases blood flow to working muscles and various organs, potentially improving the distribution and uptake of circulating nanoparticles (Lima et al., 2024). Exercise also temporarily increases cell membrane permeability and upregulates numerous transporters, which could facilitate nanoparticle entry into target tissues (Lima et al., 2024; Nanda and Yi, 2024). Furthermore, the synergistic interaction between exercise and nanomedicine is particularly relevant for neuromuscular diseases, such as Duchenne muscular dystrophy (DMD), where promoting muscle regeneration and enabling gene editing are critical therapeutic goals. Muscle training acts as a potent stimulus for myogenic processes, activating satellite cells and enhancing muscle tissue repair, which creates a biologically active niche for therapeutic intervention. Crucially, mechanical stimuli from exercise—particularly stretching—impact the integrity and mechanics of the myonuclear envelope via the LINC complex (Linker of Nucleoskeleton and Cytoskeleton). Improving the mechanical stability of the nuclear membrane may facilitate the nuclear translocation of nanoparticle-mediated gene therapies (e.g., CRISPR/Cas9 delivery systems). By combining mechanical conditioning with nuclear-targeted nanoparticles, it may be possible to overcome the significant barrier of nuclear entry, thereby enhancing the efficacy of novel gene-targeted therapies for neuromuscular genetic disorders (Donnaloja et al., 2019; Elosegui-Artola et al., 2017).

Molecular synergies may also exist between exercise-induced signaling pathways and nanoparticle actions. For instance, exercise activates antioxidant defense systems while simultaneously generating reactive oxygen species (ROS) as metabolic byproducts. Redox-active nanoparticles could complement this process by providing additional antioxidant protection precisely when endogenous systems are challenged (Lima et al., 2024).

Beyond athletic performance enhancement, exercise-nanoparticle synergies hold promise for various therapeutic applications:

1. Neurodegenerative disease prevention and treatment: Combining exercise-induced neurogenesis with nanoparticle-mediated delivery of neuroprotective compounds could provide enhanced protection against conditions like Alzheimer’s disease (Clemente-Suárez et al., 2025).
2. Targeted muscle recovery and adaptation: Nanoparticles could deliver anti-inflammatory or growth-promoting agents directly to exercised muscles, potentially accelerating recovery and adaptation processes (Lima et al., 2024).
3. Metabolic disorder management: The combined effects of exercise on insulin sensitivity and metabolic health could be augmented by nanoparticle-delivered compounds that further improve glucose regulation or fat metabolism.
4. Cognitive enhancement: Exercise-induced increases in brain plasticity might create optimal conditions for nanoparticle-delivered cognitive enhancers to exert maximal effects on memory and learning (Clemente-Suárez et al., 2025).

## Current limitations and future direction

The most significant limitation of current research is that all studies exploring nutritional nanocompounds for exercise performance have been conducted exclusively in animal models (mice and rats) (Lima et al., 2024). This restricts the direct applicability of findings to human performance and necessitates careful human trials before drawing definitive conclusions about efficacy and safety in athletic or therapeutic contexts.

Additionally, the relatively small number of studies in this field limits our understanding of the optimal nanoparticle types, dosages, timing protocols, and exercise modalities for maximizing synergistic benefits. Most investigations have focused on endurance exercise, with limited exploration of resistance training or high-intensity interval protocols that might yield different interaction patterns with nanoparticle interventions.

Nanoparticles are a broad class of materials with diverse properties, such as shape, size, chemical composition, and surface charge. The article may not fully address the variability in nanoparticle formulations and how different characteristics might influence the effectiveness of the synergy with exercise. This heterogeneity makes it challenging to generalize the findings to all NPs types.

Many of the studies that have been cited are short-term, and there is a lack of information regarding the effects that combining nanoparticles and exercise will have on human health over the long term. The evaluation of potential chronic side effects, toxicity, or any changes in the body’s response over time requires the conduct of studies that are conducted over an extended period of time.

Several promising research directions could advance our understanding of exercise-nanoparticle synergies:

1. Human clinical trials evaluating the safety, efficacy, and optimal protocols for nutritional nanocompounds in exercise contexts.
2. Investigation of potential synergies between exercise-induced neuroplasticity and nanoparticle-mediated drug delivery for treating neurodegenerative conditions.
3. Development of bioengineered wearable technologies that could monitor physiological responses to combined exercise and nanoparticle interventions in real-time.
4. Exploration of gender-specific responses to nanoparticle and exercise interventions, given the documented differences in how men and women respond to both exercise and nutritional interventions.
5. Long-term studies examining potential chronic adaptations and safety profiles of repeated nanoparticle administration in conjunction with regular exercise programs.

## Conclusion

The field investigating the synergistic effects of exercise and nanoparticles is a promising area in sports science and therapeutic applications. Current evidence, primarily derived from animal studies, indicates that nutritional compounds delivered through nanoparticle carriers may enhance exercise performance via several physiological mechanisms. These include improved antioxidant activity, reduced inflammation, and preservation of muscle integrity, enhanced mitochondrial function, and optimised availability of energy substrates.

Exercise concurrently establishes beneficial physiological conditions that may improve nanoparticle delivery and effectiveness by enhancing blood flow, altering membrane permeability, and activating complementary molecular pathways. The bidirectional relationship indicates that well-designed exercise-nanoparticle interventions may provide advantages exceeding those of either method independently.

On the other hand, there are still significant research gaps, particularly with regard to human applications, optimal protocols, long-term adaptations, and safety profiles. Investigations of these factors across a variety of exercise modalities and nanoparticle formulations should be given priority in future research. These studies should be well designed and conducted on humans. Furthermore, the investigation of gender-specific responses and the development of integrated biomonitoring approaches have the potential to further enhance our comprehension of these synergistic effects.

This field could revolutionise athletic performance enhancement and treatment of neurodegenerative, metabolic, and inflammatory diseases. Exercise science and nanotechnology may combine to create personalised, effective interventions that improve health and performance in diverse populations.
